# A scoping review of training and deployment policies for human resources for health for maternal, newborn, and child health in rural Africa

**DOI:** 10.1186/1478-4491-12-72

**Published:** 2014-12-16

**Authors:** Gail Tomblin Murphy, Fastone Goma, Adrian MacKenzie, Stephanie Bradish, Sheri Price, Selestine Nzala, Annette Elliott Rose, Janet Rigby, Chilweza Muzongwe, Nellisiwe Chizuni, Amanda Carey, Derrick Hamavhwa

**Affiliations:** WHO/PAHO Collaborating Centre on Health Workforce Planning and Research, Dalhousie University, 5869 University Avenue, Halifax, Nova Scotia B3H 4R2 Canada; School of Nursing, Dalhousie University, 5869 University Avenue, Halifax, Nova Scotia B3H 4R2 Canada; School of Medicine, University of Zambia, Nationalist Road, U.T.H., PO Box 50110, Lusaka, Zambia; Centre for Primary Care Research, University of Zambia School of Medicine, Nationalist Road, U.T.H., PO Box 50110, Lusaka, Zambia; Zambia Forum for Health Research, Post-Net Box 261, 23 Chindo Road, Woodlands, Lusaka, Zambia

**Keywords:** Africa, Child, Deployment, Human resources for health, Maternal, Newborn health, Policy, Rural and remote health care, Training

## Abstract

**Background:**

Most African countries are facing a human resources for health (HRH) crisis, lacking the required workforce to deliver basic health care, including care for mothers and children. This is especially acute in rural areas and has limited countries’ abilities to meet maternal, newborn, and child health (MNCH) targets outlined by Millennium Development Goals 4 and 5. To address the HRH challenges, evidence-based deployment and training policies are required. However, the resources available to country-level policy makers to create such policies are limited. To inform future HRH planning, a scoping review was conducted to identify the type, extent, and quality of evidence that exists on HRH policies for rural MNCH in Africa.

**Methods:**

Fourteen electronic health and health education databases were searched for peer-reviewed papers specific to training and deployment policies for doctors, nurses, and midwives for rural MNCH in African countries with English, Portuguese, or French as official languages. Non-peer reviewed literature and policy documents were also identified through systematic searches of selected international organizations and government websites. Documents were included based on pre-determined criteria.

**Results:**

There was an overall paucity of information on training and deployment policies for HRH for MNCH in rural Africa; 37 articles met the inclusion criteria. Of these, the majority of primary research studies employed a variety of qualitative and quantitative methods. Doctors, nurses, and midwives were equally represented in the selected policy literature. Policies focusing exclusively on training or deployment were limited; most documents focused on both training and deployment or were broader with embedded implications for the management of HRH or MNCH. Relevant government websites varied in functionality and in the availability of policy documents.

**Conclusions:**

The lack of available documentation and an apparent bias towards HRH research in developed areas suggest a need for strengthened capacity for HRH policy research in Africa. This will result in enhanced potential for evidence uptake into policy. Enhanced alignment between policy-makers’ information needs and the independent research agenda could further assist knowledge development and uptake. The results of this scoping review informed an in-depth analysis of relevant policies in a sub-set of African countries.

## Background

The Millennium Development Goals (MDGs), released in 2000, are considered an international blueprint for meeting the needs of the world’s most vulnerable people by 2015 [1]. The health and well-being of mothers, newborns, and children, as detailed by MDGs 4 and 5, are at the forefront of many related policy and planning discussions. The global maternal mortality rate (MMR) and child mortality rate have been halved since 1990; however, the MMR in developing areas is 15-fold of that in developed regions and four out of five child deaths under the age of five occur in sub-Saharan Africa and Asia [2, 3]. Persisting challenges in meeting MDGs 4 and 5 in 2015 and beyond are detailed in progress reports, particularly those from many African countries [1]. The MMR in sub-Saharan Africa currently stands at 510 deaths per 100,000 live births, with only 53% of deliveries attended by a skilled health provider [3]. While the birth rate and under-five population are expected to substantially increase in the sub-Saharan region, the child mortality rate (98/1,000 live births) is 2-fold that of the region with the second highest rate, and 16-fold that of developed regions [3]. These staggering numbers are related to pervasive inequities related to broader health and social system issues as well as the stark geographic mal-distribution of health services [4]. Analysis of the current state of maternal, newborn, and child health (MNCH) in Africa reveals a need for enhanced access to primary health care (PHC), emergency services, reproductive health, and family planning. Critical to achieving such enhanced access is the availability, and equitable deployment, of sufficient numbers of adequately trained human resources for health (HRH) to deliver those services [5]. The body of evidence directly linking the increased availability of skilled health providers to improved MNCH outcomes is growing [6–8]. When HRH density increases, so do important MNCH interventions – such as measles immunization and births attended by skilled personnel – which result in increases in maternal, newborn, and child rates of survival [8].

Africa faces a long-standing, unprecedented HRH crisis. Thirty-six countries in the region – the bulk of the continent – have less than the World Health Organization (WHO)’s minimum recommended density of HRH to provide basic health care to their populations [9]. Estimates suggest that nearly 1,000,000 additional personnel are needed to bring Africa up to the minimum WHO-recommended density of 2.3 doctors, nurses, and midwives per 1,000 population [9].

Despite successive resolutions over the last 20 years by the WHO Regional Committee for Africa to expand the continent’s health workforce, Africa’s regional HRH density actually declined between 2005 and 2010 [9]. Rural areas, which tend to face the largest disparities between population health needs and the necessary HRH and other resources to address them, shoulder the burden of the HRH crisis disproportionately [10]. Underscoring the importance of effective policies for the planning and management of scarce HRH are institutions hindered by poor physical infrastructure, shortages of qualified faculty, and lack of external accreditation, all of which persist despite widespread emphasis on scaling up HRH training as part of broader health system strengthening [11].

In most health systems, expenditure on HRH accounts for approximately 70% of recurrent spending [12]. Therefore, management of the investment that is HRH can have significant implications for those systems. While a number of different dimensions influence how a country’s HRH are planned and managed, perhaps the most critical are how HRH are trained and deployed [13–15]. Although information on different countries’ HRH policies and practices exists, gathering and reviewing relevant evidence is often beyond the time and resources of many country-level policy makers [16]. In an effort to synthesize existing evidence to inform HRH policy and practice, a systematic scoping review and in-depth analysis of available peer- and non-peer-reviewed literature, as well as unpublished policy documents, on the training and deployment of doctors, nurses, and midwives for maternal-child care in rural Africa, was completed. This paper presents the findings from the abovementioned scoping review, whose purpose was to identify and classify publicly accessible evidence on policies for the training and deployment of doctors, nurses, and midwives in rural Africa for promoting MNCH [17].

## Methods

In order to rapidly review the large and complex body of literature that was anticipated to exist for the training and deployment of doctors, nurses, and midwives for rural MNCH in Africa, a scoping method was adopted. This method allows for large-scale accumulation of literature, and mapping of the evidence therein, without discrimination based on methodological criteria to determine the extent of the research themes and gaps requiring additional research [18, 19]. As there is no universally-agreed upon scoping methodology, the specific two-part review strategy for this study was developed in consultation with an international Advisory Group (AG) of HRH and health experts, as well as an information scientist. Inclusion criteria were developed in collaboration with the AG and included as Table 1. The literature identified through the scoping was used to create a narrative of the relevant, available evidence, including the associated gaps. This review is not intended as a rigorous analysis of the identified studies, or the policies they address; the information provided herein provides a synopsis of the scoping component of the study. However, the scoping review results reported here were used as a preliminary step to inform an in-depth policy analysis for a sub-set of eight African countries: Ethiopia, Ghana, Mali, Mozambique, Niger, Tanzania, Uganda, and Zambia. The methods and results of that analysis are provided in the full report [17].

**Table 1:** **Inclusion criteria for peer- and non-peer reviewed literature scoping**

Aspect	Criteria
Language of publication	English, French, or Portuguese
Years published	1990–2013
Type of policy initiative	Applied (i.e., not theoretical, but some evidence that the policy has been/continues to be implemented)
Country or countries of focus	Any African country whose official languages include English, French, and/or Portuguese
Policy focus	Training and/or deployment of providers as it pertains to rural health
Type(s) of providers	Doctors, nurses, and/or midwives
Specific clinical focus	Maternal-child health: reproductive health, pregnancy, birth, newborns, childhood disease, and adolescents
Jurisdictional focus	International or national (e.g., not provincial or district-specific)
Types of data sources	Policy documents, policy evaluations, professional protocols/clinical guidelines, literature reviews, peer-reviewed research related to policy implementation and/or evaluation

### Peer-reviewed literature

Searches of the following online databases were conducted: PubMed, CINAHL, EconLit, PsychArticles, PsychInfo, Informa Health Care e-books, the Cochrane Library, ABIinform, Web of Knowledge, PAIS, JSTOR, Business Source Complete, ERIC, and EMBASE. The following key words were identified and used in various combinations with Boolean operators (and, or, not): health care delivery, health planning, health policy, policy, population health care needs, health workforce, health human resources, care providers, manpower, personnel, nurses, doctors, midwives, shortage, turnover, deployment, regulation, training, education, incentives, recruitment, retention, attrition, maternal, newborn, child, infant, adolescent, maternal-child care, rural, isolated, low resource, Africa, developing country, low income country, middle income country. Where available and appropriate, MeSH terms wildcards and explosion search strategies (sub-terms and derivatives) were used.

The content of potentially relevant articles was mapped using a data extraction tool as adapted from Price [20] and informed by the Critical Appraisal Skills Programme checklist [21]. Specifically, data for country of focus, document type, policy initiative, jurisdictional focus, provider type, and policy nature were collected. Once mapping was completed, initial exclusions of citations were made if they were not available in full text, published prior to 1990, and did not refer to an African country whose official national languages include English, French, and/or Portuguese. Ethiopia was the only exception; although the country’s official language is Amharic, Ethiopia publishes many of its health policy documents in English, and was also identified by the AG as a unique case for consideration due to its achievement of MDG 4 in 2013 [22].

Additional articles were identified for consideration by the Zambian research team as well as the AG based on their personal familiarity with particular African countries. Research team members from Zambia and Canada then pooled these citations with those found in the database searches. From this pool, articles were subjected to an initial abstract review to remove those that clearly did not meet the inclusion criteria. Three members from the research team reviewed the title, abstract, and full text, independently applying the inclusion criteria (Table 1), based on the research questions, for the remaining articles. Inconsistencies in reviewers’ inclusion and exclusion decisions were resolved through group deliberation until consensus was reached. The final body of publications was analyzed and synthesized using content and thematic analyses.

### Non-peer-reviewed literature

#### Phase I

For each country in the designated linguistic groups, a directed search of websites operated by ministerial bodies responsible for health planning and policy was conducted. The search engine Google was used to locate such websites, which were then navigated by the tabs and menus (i.e., policies, publications, legislation, guidelines, etc.) available on the homepage. The availability of relevant documents from Ministry of Health (MoH) websites varied considerably. Documents were scanned and pulled as guided by the inclusion criteria (Table 1).

If not available on the ministerial websites, targeted internet searches were used and direct requests made to the AG in an attempt to obtain copies of each country’s National Health Policy, National Strategic Health Plan, National Strategic Plan for Human Resources for Health, and/or related documents, if available.

#### Phase II

Websites of relevant professional associations, research networks, and international and national non-governmental organizations were selected (Table 2), in consultation with the AG, for further non-peer reviewed literature searching. Based on the individual navigability of each website, unique search strategies were developed; either commencing with topic-relevant tabs (i.e., MNCH, HRH, education, training and deployment, health workforce, policy, etc.) or country tabs.

**Table 2:** **Websites of professional associations, research networks, and international and national non-governmental organizations used in scoping review**

Name	Website
African Centre for Global Health and Social Transformation (ACHEST)	http://www.achest.org
African Health Workforce Observatory	http://www.hrh-observatory.afro.who.int/en/home.html
Bill and Melinda Gates Foundation	http://www.gatesfoundation.org/
Eldis	http://www.eldis.org
Global Health Workforce Alliance	http://www.who.int/workforcealliance/en
The Health Policy Monitor	http://www.hpm.org
Health Professionals for a New Century	http://www.healthprofessionals21.org/
HRH Global Resource Center	http://www.hrhresourcecenter.org
International Confederation of Midwives	http://www.internationalmidwives.org
	http://www.internationalmidwives.org/global/french
International Council of Nurses	http://www.icn.ch/en
	http://www.icn.ch/fr
International Federation of Gynecology and Obstetrics	http://www.figo.org
Institute for Education Sciences (ERIC)	http://www.eric.ed.gov
Medicus Mundi	http://www.medicusmundi.org/en
	http://www.medicusmundi.org/fr
Regional East African Community Health (REACH) Policy Initiative	http://www.health.eac.int
SUPPORT: Supporting Policy Relevant reviews and Trials	http://www.support-collaboration.org/
THET Partnerships for Global Health	http://www.thet.org/
The World Bank	http://www.worldbank.org/en/topic/health/brief/human-resources-health
WHO Collaborating Centre – University of Western Cape	http://www.hrhforafrica.org.za.
WHO Regional Office for Africa	http://www.afro.who.int/en/clusters-a-programmes/hss/cluster.html

## Results

### Peer-reviewed literature

**Figure 1:**
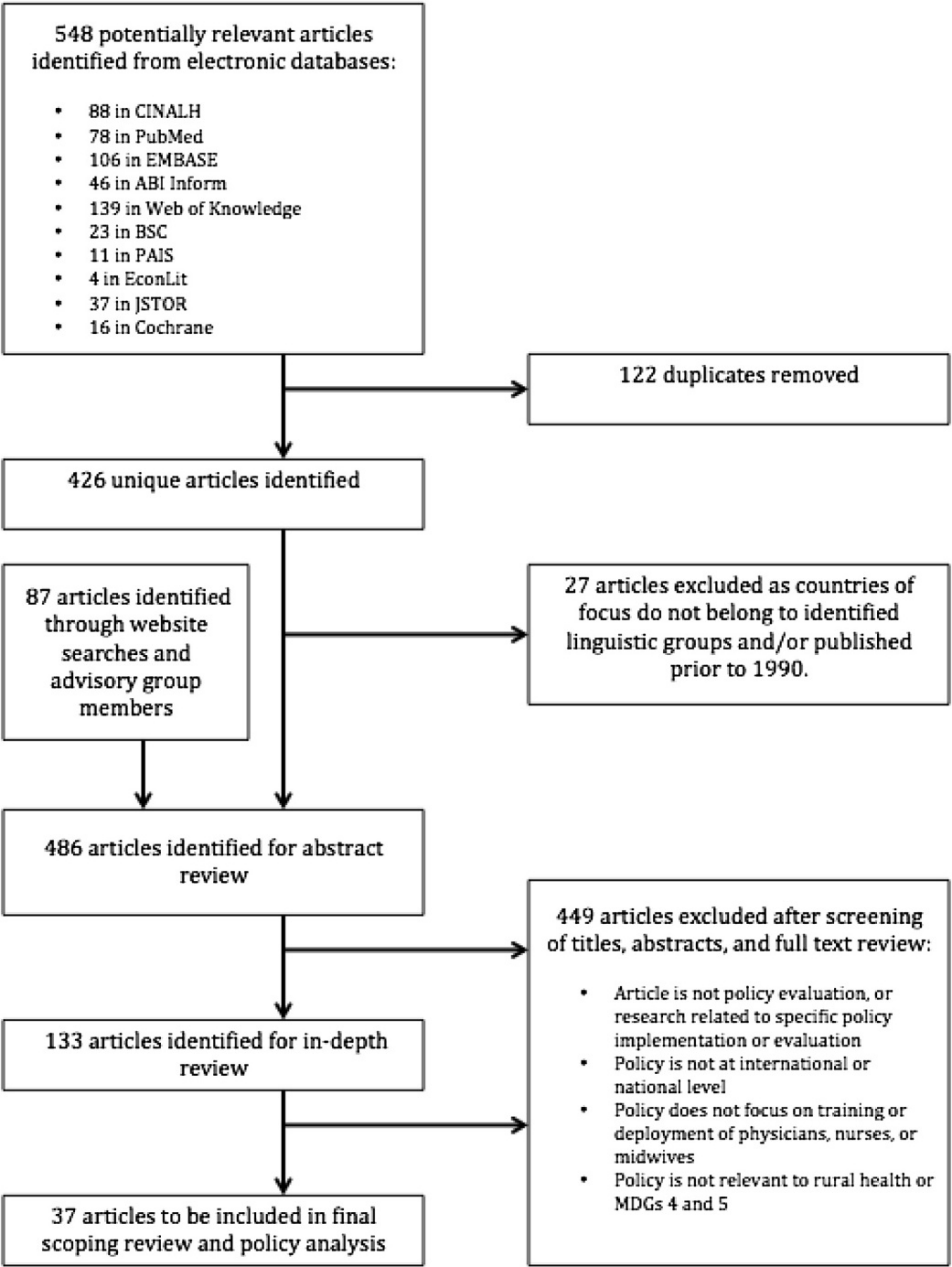
**Scoping review results.**

**Figure 2:**
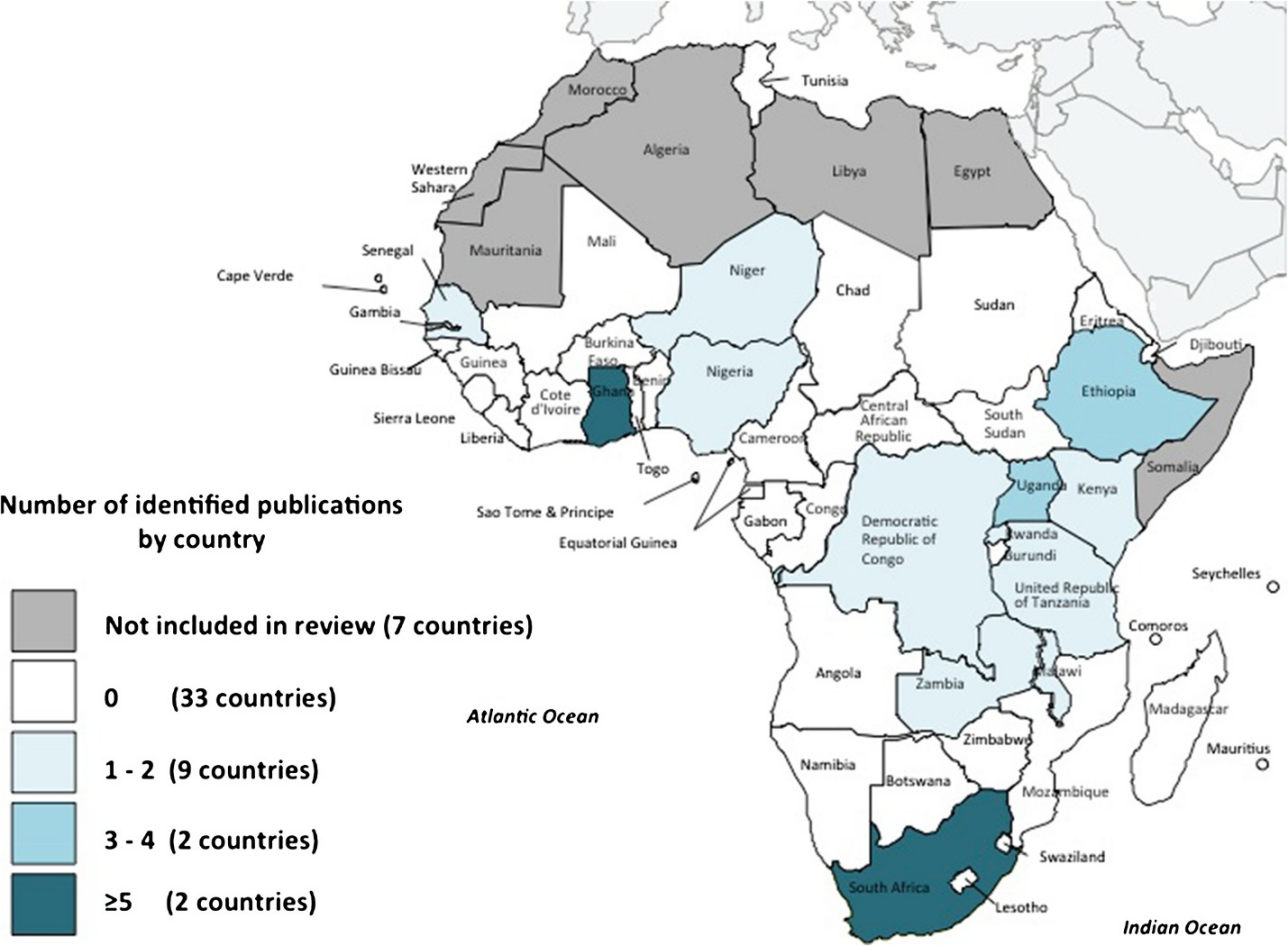
**Number of peer-reviewed articles by country.**

The scoping of electronic databases returned a total of 548 peer-reviewed articles, including 122 duplicates. The 426 unique articles were combined with the 87 additional articles identified by the Zambian research team and AG members, totalling 513 articles to be reviewed. Of these articles, 37 met the inclusion criteria (Figure 1).Articles meeting the inclusion criteria covered 13 of a possible 46 countries included in the review, representing each region of Africa, as well as all designated linguistic groups (Figure 2). With nine peer-reviewed articles, Ghana had the highest representation, followed by South Africa and articles addressing multiple nations, each with five. Ethiopia had four articles, and the remaining 10 countries were represented by one to three publications each.

**Figure 3:**
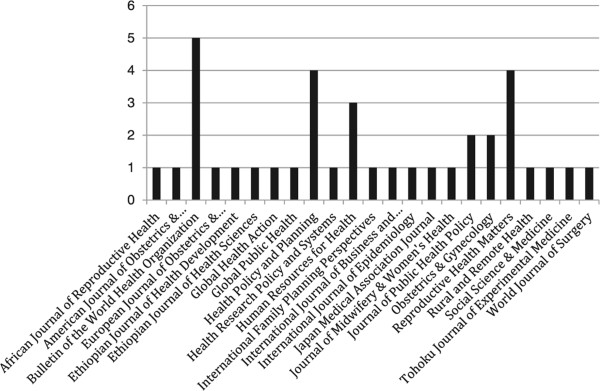
**Contributing source journals for peer-reviewed articles.**

**Figure 4:**
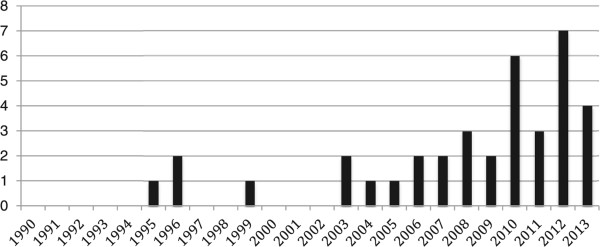
**Peer-reviewed articles by year of publication.**

The identified peer-reviewed articles were published in 22 unique journals (Figure 3). The *Bulletin of the World Health Organization* (3 papers), *Health Policy and Planning* (4), *Reproductive Health Matters* (4), and *Human Resources for Health* (3) were the most frequent contributors. The distribution of articles by year of publication is shown in Figure 4, and demonstrates that the vast majority (89%) of the peer-reviewed articles were published since 2003.

There was equal representation of all professions of focus – doctors, nurses, and midwives – in the identified literature. However, many of the selected articles implicitly included all health professions due to the high-level nature of the policies explored, such as those pertaining to health sector reforms and national health policies. Applicability to rural MNCH similarly ranged from explicit to implicit, based on the level of the policy. Policies that focused exclusively on training or deployment were limited, with the majority of the literature addressing both training and deployment, either directly or as embedded components of broader policies.

A cursory review of excluded policies not meeting all inclusion criteria provided insight into a diverse range of other research and initiatives related to training and deployment of HRH for improved rural MNCH, e.g., i) theoretical policies evaluated using various modelling techniques, such as the Markov models for South African nursing policies, and discrete choice experiments exploring for example how to make rural jobs more attractive to health workers in Tanzania and midwives in Ghana [23–25]; ii) pilot programs designed, conducted, and evaluated for potential scale-up such as the WHO safe childbirth checklist and facility-based multifaceted interventions on obstetrical care quality in Mali and Senegal [26, 27]; iii) literature reviews conducted to take inventory of what is known and which knowledge gaps existed about, for example, the role of private sector in producing nurses in multiple nations, including Kenya and South Africa [28]; iv) in-depth situational analyses to better inform policy priorities [29, 30]; and v) the use of providers outside of our scope such as the use and deployment of community health workers in sub-Saharan Africa and training of traditional birth attendants [31, 32].

A synthesis of policy information provided exclusively from the content of the peer-reviewed literature, classified by policy foci (e.g., training only, training and deployment, deployment only, recruitment and retention, etc.) is provided below, including any available information on the impacts of these policies. A summary is provided in Table 3.

**Table 3:** **Summary of policies information from peer-reviewed publications**

Policy type	Name	Country	Provider(s)
**Training**	Localized obstetric and gynaecology training	Ghana	Doctors
	Life-saving skills	Nigeria	Midwives
	Integrated management of childhood illness	Uganda	Doctors, nurses, midwives
**Deployment**	Rural allowance policy	South Africa	Doctors, nurses
	Occupation-specific dispensation incentive strategy	South Africa	Nurses
	Financial incentive scheme	Niger	Doctors
	Medicalization of rural areas	Mali	Doctors
	Emergency hiring plan	Kenya	Doctors, nurses
	Plan Cobra	Senegal	Doctors, nurses, midwives
	Surgical camps	Uganda	Doctors
	Community operation theatres	Uganda	Doctors
**Training & Deployment**	Emergency human resources program (supporting the Essential Health Package)	Malawi	Doctors, nurses
	10 year Strategic Plan for HRH	Zambia	Doctors, nurses, midwives
	Rurally located medical schools	Democratic Republic of the Congo	Doctors
	Two to four year compulsory service program (CSP) with graded salary, preference for post-grad (PG) specialization, textbooks, computer, degree, variable length of service incentives	Ethiopia	Doctors
	One to three year CSP with PG specialization and scholarships incentives	Ghana	Doctors, nurses
	Three year CSP with PG specialization and scholarships, career advancement incentives	Kenya	Doctors, nurses
	One year CSP with graded salaries and housing incentives	Lesotho	Doctors
	Two year CSP with housing and career advancement incentives.	Mozambique	Doctors, nurses, midwives
	Two year CSP with graded salary and PG scholarship incentives	Namibia	Doctors
	One year CSP with license, PG specialization, and career advancement incentives	Nigeria	Doctors, nurses, midwives
	One year CSP with graded salary and license to practice in private sector incentives	South Africa	Doctors
	Three year CSP with graded salary, housing, child education, loads, preference for PG specialization and scholarship incentives	Zambia	Doctors
	Three year CSP with license to practice and preference for PG specialization incentives	Zimbabwe	Doctors
**Embedded**	Decentralization	Ghana	Implicitly all
	Decentralization	Ethiopia	Implicitly all
	Decentralization by devolution	Tanzania	Implicitly all
	Sector wide approach	Tanzania	Implicitly all
	Millennium rural initiative	Ethiopia	Implicitly all
	Rural health improvement program	Niger	Implicitly all
	Child survival strategy	Uganda	Implicitly all
	Accelerating the reduction of maternal and neonatal mortality	Uganda	Implicitly all
	Sexual and reproductive health policy	Rwanda	Implicitly all
	Facility-based childbirth policy	Rwanda	Implicitly all
	Reproductive health service policy and standards	Ghana	Doctors, nurses, midwives
	Cervical screening policy	South Africa	Nurses
	Choice on termination of pregnancy act	South Africa	Doctors, nurses, midwives

### Policy information from peer-reviewed literature

#### Training

The Ghanaian government adopted a retention-focused training program into its HRH policy in recognition of its success, committing to its sustainability through administrative and funding assistance [33, 34]. The local training program for obstetrics and gynaecology specialists, launched initially by a partnership between Ghanaian medical schools, the United Kingdom’s Royal College of Obstetricians and Gynecologists, and the American College of Obstetricians and Gynecologists, aimed to counteract the expatriation of internationally-trained Ghanaian providers. Thirty-seven of the 38 specialists who successfully completed this program from its initiation in 1989 to 2006 remained in Ghana to practice, the majority practicing in the public sector [35]. Additional research on the same program described the high calibre of specialists produced, the innovative community-based curriculum approach, a favourable cost-benefit evaluation, a reduction in MMR at host hospitals, and evidence that the trainee’s service and ability to build broad health sector capacity is retained if educated domestically [33, 34].

In a move to strengthen its commitment to the first level of referral, the Nigerian MoH adopted modified and expanded training strategies. Midwifery competency upgrading through Life Saving Skills training – a ten-module program aimed at management of emergency obstetric conditions – was paired with interpersonal skills training across provider groups and provision of necessary equipment and supplies. This was found to result in large gains for MNCH seen in the reduction of post-partum haemorrhage, prolonged labour, and stillbirths [36].

Uganda, too, sought to strengthen its primary level of referral capacity by training qualified staff (nurses, midwives, and doctors, when available) in the Integrated Management of Childhood Illness (IMCI) program between 2000 and 2002 [37]. Training efforts were then scaled up through a modified IMCI course for auxiliary staff. Although both “qualified” and “auxiliary” staff were found to perform significantly better than those who did not receive training, outcomes associated with IMCI were not raised to an acceptable level through this initiative [37]. For example, following the completion of IMCI training, only approximately half of children identified as having malaria or pneumonia received treatment that was correct and complete. Clear communication and/or delivery of the home care messages to the caretakers of the unwell children was lacking; only 68% of caretakers received instructions for home treatment, and of those less than 35% were informed of the IMCI home care messages. The researchers attributed these shortcomings to the influence of contextual factors such as the lack of supervision, supportive work environments, adequate supplies of medical technology, and complimentary policies [37].

#### Deployment, recruitment, and retention

Two strategies that South Africa has employed for deployment and retention of HRH, with implications for rural MNCH, were described as having significant weaknesses. The 2004 Rural Allowance policy was intended to address the critical maldistribution of HRH by attracting a health workforce to underserved areas through allowances equal to 18 to 22% and 8 to 12% of salaries for doctors and nurses, respectively [38]. Upon qualitative evaluation, through key-stakeholder interviews, this policy demonstrated a lack of evidence-based design and seemed to have sub-optimal effects on the unequal distribution. For example, as the allowance was not graded according to degree of ruralness, the gains were the same if nurses chose to take posts in rural towns or deep rural areas; preference, in the majority, was shown for the former. Additionally, financial incentives were not deemed sufficient to retain the relocated staff. After a period of time, the prioritization of increased income seemed to give way to the need for non-financial incentives such as access to quality education for children and professional development. Poor communication and definition of implementation parameters (i.e., which providers were eligible for the allowance and why, what qualified as a rural zone) and an absent monitoring and evaluation mechanism hindered the program further [38]. The 2007 implementation of the Occupation-Specific Dispensation Incentive strategy, a financial incentive scheme for the retention of nurses in the public sector (including rural health facilities), was described as being pushed forward prior to many preconditions being met, such as gathering of complete and accurate specialized nursing registration data from the South African Council of Nurses [39]. Identified as further undermining the process was too little consideration given to the resources, relationships, and communication that were necessary for success [39, 40].

Additionally, an international review of evaluated recruitment and retention schemes indicated only by name the presence of a financial incentive scheme in Niger targeted at doctors, pharmacists, and dental surgeons, and a “medicalization of rural areas” program in Mali [41]. However, as there was minimal information available about these policies other than their names, it is not possible to discern what their intended impacts or considerations may have been for MNCH in rural areas.

Several temporary employment contracting systems, characterized by fixed terms, locations, and roles, with the option of renewal, were described as increasing deployment in several countries. Kenya’s Emergency Hiring Plan, initially managed by the private sector and later by the national government, deployed 830 new health staff to 219 public health facilities over a 6-month period in 2007 [42]. The same plan resulted in the recruitment of 1,836 additional nurses between 2005 and 2009. This was most beneficial in the North Eastern province, comprising some of the most remote areas in the country, saw a 37% increase in nurse density [43]. In Senegal, 365 new HRH contracts issued between 2006 and 2008, and the re-opening of 122 health posts have been attributed to Plan Cobra, a contracting method which allowed for short-term employment while still being eligible for the benefits associated with traditional recruitment through the Ministry of Public Service [44].

Uganda – to address the imbalance of having 90% of its doctors located in Kampala, where only 10% of its population lives – opted for an array of policies with varying timelines: a specialist outreach program, rural surgical camps, and building operating theatres at the sub-district level [45]. Of these, only the surgical camps and development of operating theatres had implications for rural MNCH. In the former, which occurred for one to two weeks once or twice annually (13 camps from 1997 to 2004, ranging from 100 to 900 operations per camp), gynaecological procedures were one of the most common. The latter was primarily motivated by the need for improved access to emergency obstetric care [45]. Both, however, struggled due to contextual issues: maintaining adequate staff in undesirable rural and remote areas, high demands on pre-existing providers, and a low flow of resources [45]. All three policies described above involved components of sensitizing communities to the programs to be implemented and training for support staff at the rural facilities.

#### Training and deployment

Policies from the scoping review frequently addressed training and deployment in tandem. Malawi’s Emergency Human Resources Program, in support of its Essential Health Package, includes five main facets: salary top-ups, developing domestic training capacity, using international volunteer HRH as a stop-gap measure, providing international technical assistance to bolster planning and management capacity, and improving monitoring and evaluation to inform short-term policy initiatives to meet long-term goals [46, 47]. A 40% increase in doctors and 30% increase in nurses in the country between 2003 and 2007 are attributed to this policy [46]. Zambia’s MoH envisioned a similar approach to addressing HRH shortages in describing improved training, deployment, and retention through its 10-year Strategic Plan for HRH [48, 49]. Specifically, it emphasizes the need and costs of increasing production of midwives to the critical quantity necessary for improving maternal mortality rates [49]. Longombe’s 2009 study in the Democratic Republic of the Congo suggested that rural training and deployment challenges can be tackled simultaneously through the use of rurally-located education; 81% of graduates from the country’s rural medical schools chose rural postings, compared to only 24% of those graduating from urban-located institutions [50].

An international review (including 11 African countries) by Frehywot et al. [51] identified multiple compulsory service programs for rural posting or retention that inherently address deployment, but also incorporated training components. These strategies involved one to four compulsory years of service, with few offering the option of a “buy-out”. The bulk of the policies applied to doctors and nurses, and all included one or more of the following incentives: licence to practice (in both public and private sectors), graded salaries, preference for post-graduate training, scholarships, career advancement, housing, child education, and medical assistance [51].

#### Embedded policies

The remaining literature explored policies not explicitly designed to address rural MNCH through training and/or deployment of the selected providers, but via embedded or implied components of policies with broader mandates. These included, for example, high-level, structural policies such as decentralization of health care planning and administration, health sector development policies and the use of a sector-wide approach for allocating donor funds [52–55].

High-level, rural-specific policies with embedded strategies for HRH deployment and implications for MNCH were also identified. Defining government and community member roles was attempted in one study to improve the implementation of The Millennium Rural Initiative in Ethiopia, which had clear implications for rural MNCH through its expansion of PHC delivery to remote areas [56, 57]. Niger’s Rural Health Improvement Program also aimed to increase PHC coverage by upgrading rural health facilities to provide PHC, and dispatching newly-trained village health teams. The facility improvements were found to lead to a significant increase in MNCH service provision and a 32% decrease in the likelihood of under-five mortality among those who lived near them compared to those who did not. Deployment of the village health teams was not linked with lowered rates of mortality [58].

Increased political prioritization of MNCH implicitly calls for similar attention to HRH issues. The 20% decrease in neonatal mortality in Uganda between 2000 and 2010 was attributed to its constellation of evolving policies – e.g., its Roadmap to Accelerating the Reduction of Maternal and Neonatal Mortality and Child Survival Strategy – many of which called for increased training and deployment of HRH to meet the strategic objectives [59]. Similar to Uganda’s broad policy response, Rwanda has implemented a Sexual and Reproductive Health Policy (under the Health Sector Policy), Facility-based Childbirth Policy, and a National Family Planning Policy, all of which require a robust HRH supply. In light of this, and the HRH shortages resulting from the 1994 genocide, Rwanda politically backed an increased production of doctors, nurses, and midwives for the public sector between 2005 and 2008; the majority were deployed to rural areas [60].

Ghana’s National Reproductive Health Service Policy and Standards, revised in 2003, reformed regulations for the provision of reproductive health services and required associated training: essential obstetric care, life-saving skills, safe motherhood, manual vacuum aspirations, and post-abortion care [61]. The scoping review identified two papers examining this policy through the lens of abortion provision. One analyzed the cost-effectiveness of post-abortion care (PAC) training and the individual levels of provision among doctors and midwives. Of the 28% of clinicians in the study providing PAC after receiving the training, 80% were doctors and 20% midwives. Although training midwives results in a lower yield of PAC – which may increase if the identified barriers to provision are addressed – than training doctors, it is suggested that midwife PAC training is an efficient and cost-effective method to reduce maternal mortality due to unsafe abortions [62]. The other applied policy theory to identify overall provider-related barriers to its implementation of the National Reproductive Health Service Policy and Standards. The researchers found that the conflict between doctors’ and midwives’ responsibilities as health providers and their personal moral and religious values was the most significant barrier to providing safe-abortion services. Although obstetricians were exposed to a greater body of medical and public health information, allowing for the tempering of religious views, the more rurally located midwives had lower access to such information and were therefore more driven by their fundamental religious beliefs [63].

Legislation in South Africa established qualified midwives as legal abortion providers; as of 2003, 135 midwives had completed training and safe abortion provision went from 714 in 1997 to 5,168 in 1999. Physical deployment was not addressed in this policy, however, it demonstrated a unique strategy of utilizing midwives, a provider with a significant pre-existing presence in rural communities [64]. Additionally, South Africa’s Cervical Screening policy mandated nurse training in Pap smear provision, again recognizing the potential of upgrading skills of previously deployed HRH [65].

### Non-peer reviewed literature

#### Phase I

At the time of scoping review, the assessed MoH websites varied widely in the functionality and availability of relevant documents. South Africa’s MoH website, Ghana’s MoH/Ghana Health Service website, and Mozambique’s Human Resources Observatory provided a wide assortment of relevant policy documents. Several other MoHs had operational websites, but had reduced functionality due to broken links, sections designated as “under construction”, and/or a lack of available policy documents. MoH websites of some countries were not located at all. Information on content, implementation, and impact of relevant policies for a selected sub-set of countries is provided in the full report [17].

#### Phase II

Scoping of the selected websites produced a wide variety of applicable literature for the country sub-set selected for the in-depth policy review following the scoping: professional guidelines and protocols, independent policy evaluations, conference notes and proceedings, and additional peer-reviewed literature. These documents, though not synthesized as part of this paper, were used to frame the issues of HRH and MNCH and to inform the country context for the in-depth policy analysis described in the full report [17] and to identify relevant policies to guide specific inquiries for additional information to the AG.

No official government policy documents that met the inclusion criteria (i.e., training and/or deployment of nurses, midwives, and doctors for MNCH in rural Africa) were available through the website scoping. Considering the narrow parameters of the research questions, this was not unexpected. Many of the official government policy documents located via directed Google searches were hosted on websites administrated by international organizations such as the United Nations Population Fund and WHO.

## Discussion

Based on the available policy documents, there were several key findings from the scoping review. First, there was an overall paucity of evidence and information on training and deployment policies for doctors, nurses, and midwives for MNCH in rural Africa. Although a multi-faceted search strategy was employed, relatively few policies meeting the specific inclusion criteria were identified. Throughout the scoping of the peer and non-peer reviewed literature, the breadth and depth of work that is being done around leveraging the health workforce for improved health in Africa was apparent. However, comparatively few documents from the original body of reviewed publications met our specific inclusion criteria. Many publications covered policies that were not at the national level, did not target rural zones, had clinical foci other than MNCH, and/or pertained to providers other than doctors, nurses, or midwives. This lack of information may indicate that such policies are scarce or non-existent in African countries, but not necessarily so; it may instead point to varying government capacity to make policies openly and publicly accessible. This is supported through the finding that while many documents were not hosted on governmental websites, they were readily located on the sites of organizations and agencies with a greater capacity for knowledge sharing.

Regardless of the cause, the lack of available evidence on HRH policy design, implementation, and impact in developing countries in Africa suggests that these have not been priority research areas. This bias was confirmed by the multi-national reviews that met the inclusion criteria for this review. All such publications (with the exception of the single review article dealing specifically with MNCH in the 68 priority MDG countdown countries [46]) had an under-representation of studies from Africa; often a factor acknowledged by the authors [36, 41, 51, 61]. Furthermore, it appears that this issue is not unique to Africa. Other developing regions, such as South East Asia, the Middle East, and South America, also suffer from a lack of research specific to the HRH policy process as it pertains to MNCH [66–68]. This reflects not only a lack of research being done where it is needed most (i.e., countries enduring acute HRH crises), but also limits the generalizability of findings to the less developed world [41, 67, 68]. The fact that the majority of peer-reviewed articles were published in 2003 or later may reflect the impact that the introduction of the MDGs in 2000 has had on priority setting for research and policy in African nations and suggests that momentum may be building for both HRH and MDGs 4 and 5 research.

Complete documentation of and/or research on any one policy, from development to implementation to impact, was only apparent if such details were included in an individual peer-reviewed article. No such understanding could be drawn from piecing together multiple articles covering the same policy, as few of the policies identified were discussed in more than one publication. Perhaps more importantly, no such understanding could be drawn from any government documents. This lack of apparent continuous evaluation of policies from development to implementation to outcomes has great implications for the establishment of an evidence-based policy process. Primarily, without on-going monitoring and evaluation, implemented policies cannot be analyzed with respect to their impact, and thus cannot be readily developed upon, improved, or scaled up with success. Secondly, having a less than comprehensive and readily available evidence base will undermine even the strongest political will to use evidence in policy. The lack of apparent evidence-based policy throughout this review underscores an issue, where research is not reflected in health policy and vice-versa, that needs to be addressed [69, 70]. Similar phenomena have been found in other reviews of training and deployment policies for HRH in rural areas [41, 66, 71]. A broad explanation as to why research has so little influence and uptake into policy is that the policy and research cycle occur at different speeds, inhibiting their ability to inform one another [72]. To help address this imbalance and increase evidence use the literature suggests personal communication and knowledge brokering, the broader use of research summaries and with policy recommendations, and timely relevance, which often requires rapid synthesis of pre-existing evidence [72–74]. This disconnect between research and its use in policy demands an improved alignment and coordination between the independent research agenda and the needs of policy makers.

Several peer-reviewed articles noted that policy execution, sustainability, and impact are defined by the context of implementation [45, 63, 65]. For example, the scaling-up of IMCI in Uganda was limited, and IMCI training alone was found not to be adequate to improve child health and that a supportive infrastructure, work environment, access to supplies, and political context were also required [37]. Nigeria, in prioritizing a contextual approach to HRH policy, implemented their Life-Saving Skills training for midwives concurrently with provision of necessary equipment, supplies, and team training. This integrated approach produced a stronger health care team, supportive management, and the identified gains in MNCH [36]. When these examples are considered together with current literature, they suggest that the consideration of context, and its use as a form of evidence, is paramount throughout the policy process and analysis [75–77].

The majority of the included primary research employed a variety of both qualitative and quantitative methods. This diversity of methods provides opportunity for triangulation of findings to inform contextual understanding of the policy process and may be helpful in developing specific recommendations for developing, implementing, and evaluating policy.

Where all included publications and policies covered doctors, nurses, and/or midwives, either explicitly or implicitly, many also addressed provider groups outside of the inclusion criteria such as clinical officers, medical assistants, laboratory staff, pharmacists, and environmental health officers [37, 45]. Many excluded documents also pertained to other professions. This underscores the importance of considering other cadres in the management of MNCH in rural settings and future scoping and policy analysis research.

Although there is an overall paucity of information on the specific types of policies sought, it is evident that attempts are being made to address MNCH in rural Africa. In addition to the increase in the body of related work since 2003, the global HRH policy and research community appear to be continuing to create a rich description of the HRH crisis in Africa. Findings from this review highlight the importance of stakeholder perspectives and consultation – such as community members, providers, and decision makers – in the design, implementation, and evaluation of policies [56, 78, 79]. If complex, comprehensive data is available to decision makers, HRH planning and health system policies are more likely to be responsive to complex contextual factors (political, economic, social, etc.).

### Limitations

Individual African country names were not included as search terms for the peer-reviewed literature. Future scoping work should include countries as keywords to identify a larger body of work. Further, given the wide range of terminology used to describe HRH policy options, the search criteria may have missed qualifying documents. Although not feasible for this study, future scoping work should involve forward and reverse citation mining to increase the number of relevant publications.

As limitations in methodology will affect the findings, caution must be taken in drawing firm conclusions from this scoping review about the quantity and quality of work being conducted in Africa related to the training and deployment of doctors, nurses, and midwives for rural MNCH. The results of the review should not be used as a proxy measure for the existence, implementation, and impact of the types of policies in question, but rather as a measure of the features and accessibility of relevant, available evidence on these types of policies.

## Conclusions

In order to improve on the current state of MNCH in rural Africa, the available health workforce needs to be effectively governed through strong policies. To inform the required political process and provide insight into existing policies to plan and manage the doctors, nurses, and midwives caring for women, newborns, and children in rural Africa, the study obtained and classified relevant documents through a scoping review. Despite a multi-faceted search strategy, relatively few policies meeting our inclusion criteria emerged. Although limited, the identified policies demonstrated a wide variety of strategies to improve rural MNCH outcomes through the deployment and training of doctors, nurses, and midwives. The relative paucity of information could be attributed to a lack of i) policies specific to this specific union of subjects; ii) prioritization of this topic in the research community; and/or iii) governmental capacity to make relevant policies publicly available. This dearth of readily accessible information has the potential to undermine the intention of decision-makers to use evidence in policy development and must be addressed. Further, it is apparent that policy development should also be informed by contextual evidence, as it can greatly affect the overall policy impact.

The results of this review provided the foundation for the second part of this study – an in-depth analysis and review of HRH deployment and training policies for MDGs 4 and 5 in a sub-set of countries in rural Africa. As identified by both the scoping and in-depth review, expansion of the methodology to include country-specific search terms, additional cadres of providers, and both qualitative and quantitative methods is necessary. Further research is required to explore the factors that influence policy, and how it supports and/or thwarts the education and deployment of HRH to care for women, newborns, and children.

## Abbreviations

AG: Advisory Group
IMCI: Integrated Management of Childhood Illness
MDG: Millennium Development Goals
MMR: Maternal Mortality Rate
MNCH: Maternal, Newborn, and Child Health
PAC: Post-abortion care
PHC: Primary Health Care
HRH: Human Resources for Health
WHO: World Health Organization
MoH: Ministry of Health.
